# Human Alzheimer’s disease gene expression signatures and immune profile in APP mouse models: a discrete transcriptomic view of Aβ plaque pathology

**DOI:** 10.1186/s12974-018-1265-7

**Published:** 2018-09-06

**Authors:** Sarah M. Rothman, Keith Q. Tanis, Pallavi Gandhi, Vladislav Malkov, Jacob Marcus, Michelle Pearson, Richard Stevens, Jason Gilliland, Christopher Ware, Veeravan Mahadomrongkul, Elaine O’Loughlin, Gonzalo Zeballos, Roger Smith, Bonnie J. Howell, Joel Klappenbach, Matthew Kennedy, Christian Mirescu

**Affiliations:** 1 In Vivo Pharmacology, Merck & Co, Kenilworth, New Jersey USA; 2 Genetics and Genomics, Merck & Co., West Point, Pennsylvania USA; 3 Neuroscience, Merck & Co., Merck Research Labs, Boston, Massachusetts USA; 4 Genetics and Genomics, Merck & Co., Merck Research Labs, Boston, Massachusetts USA; 5 Neuroscience, Merck & Co, West Point, Pennsylvania USA; 6 Systems Toxicology, Merck & Co., Kenilworth, New Jersey USA; 7 Infectious Diseases and Vaccines, Merck & Co., West Point, Kenilworth, Pennsylvania USA

**Keywords:** Alzheimer’s disease, Transcriptomics, Plaque, Microglia, Neuroinflammation

## Abstract

**Background:**

Alzheimer’s disease (AD) is a chronic neurodegenerative disease with pathological hallmarks including the formation of extracellular aggregates of amyloid-beta (Aβ) known as plaques and intracellular tau tangles. Coincident with the formation of Aβ plaques is recruitment and activation of glial cells to the plaque forming a plaque niche. In addition to histological data showing the formation of the niche, AD genetic studies have added to the growing appreciation of how dysfunctional glia pathways drive neuropathology, with emphasis on microglia pathways. Genomic approaches enable comparisons of human disease profiles between different mouse models informing on their utility to evaluate secondary changes to triggers such as Aβ deposition.

**Methods:**

In this study, we utilized two animal models of AD to examine and characterize the AD-associated pathology: the Tg2576 Swedish APP (KM670/671NL) and TgCRND8 Swedish plus Indiana APP (KM670/671NL + V717F) lines. We used laser capture microscopy (LCM) to isolate samples surrounding Thio-S positive plaques from distal non-plaque tissue. These samples were then analyzed using RNA sequencing.

**Results:**

We determined age-associated transcriptomic differences between two similar yet distinct APP transgenic mouse models, known to differ in proportional amyloidogenic species and plaque deposition rates. In Tg2576, human AD gene signatures were not observed despite profiling mice out to 15 months of age. TgCRND8 mice however showed progressive and robust induction of lysomal, neuroimmune, and ITIM/ITAM-associated gene signatures overlapping with prior human AD brain transcriptomic studies. Notably, RNAseq analyses highlighted the vast majority of transcriptional changes observed in aging TgCRND8 cortical brain homogenates were in fact specifically enriched within the plaque niche samples. Data uncovered plaque-associated enrichment of microglia-related genes such as ITIM/ITAM-associated genes and pathway markers of phagocytosis.

**Conclusion:**

This work may help guide improved translational value of APP mouse models of AD, particularly for strategies aimed at targeting neuroimmune and neurodegenerative pathways, by demonstrating that TgCRND8 more closely recapitulates specific human AD-associated transcriptional responses.

**Electronic supplementary material:**

The online version of this article (10.1186/s12974-018-1265-7) contains supplementary material, which is available to authorized users.

## Background

Alzheimer’s disease (AD) is a progressive, neurodegenerative disease characterized by a wide spectrum of pathological hallmarks including amyloid-beta (Aβ) plaques, intraneuronal neurofibrillary tangles, atrophy of synapses, and dendritic arbors, with associated cognitive decline [1]. While histological studies have characterized the morphology and process complexity of glial cells in a wide-range of neurodegenerative diseases, specific genomic and proteomic data regarding how microglia and astrocytes are modulated by disease, and vice versa, remain unknown. The lack of data regarding microglial reactivity in neurodegeneration is somewhat due to controversy regarding the utility of specific AD mouse models as windows into or prototype for human disease, and a paucity of studies using panoramic unbiased molecular profiling approaches to better describe local pathological changes occurring within the Aβ niche.

Numerous studies using mouse AD models have identified and pre-clinically validated mechanisms targeted at either neuro-inflammatory or innate immune pathways [2–5]. However, the predictive value of these data is predicated on the translational relevance of the selected mouse model. Hence, clear understanding of how molecular pathways, particularly those related to the neuroimmune response, change with respect to disease pathology in these models is a key to accurate data analysis and interpretation. RNAseq is well suited to mapping out transcriptome changes across the entire genome and has significant advantages to low-throughout platforms and microarrays which are biased to known transcripts and generally remain limited to comparative inferences. One goal of the current study was to complete longitudinal evaluation of two different APP mouse models using RNAseq in brain regions demonstrating heavy plaque burden to evaluate their overlap with gene expression signatures previously identified in human AD brain samples.

Microglia are the brain’s resident immune cells that maintain central nervous system (CNS) homeostasis, constantly survey their environment, and react to injury by initiating an inflammatory reaction [6]. They express a diverse set of pattern recognition receptors capable of sensing these damage-associated signals and pathogens in the extracellular milieu and respond to neuronal injury and tissue pathogens by rapidly extending processes toward and chemotaxing into the pathological niche. Not surprisingly, in the AD brain, microglia cluster and surround Aβ plaques, a phenomenon also noted in many mouse models [7–9]. Despite the similar observations between mouse and humans, it remains to be seen how the plaque niche in AD mouse models may be dysregulated with regard to expression of human AD signatures, inflammatory versus innate immune pathways, and what transcriptome-wide profiling insights can be gained about biological changes occurring in the plaque-associated tissue. Notably, drugs targeting inflammation in AD, such as COX inhibitors and nonsteroidal anti-inflammatory drugs (NSAIDs), have failed in the clinic underscoring the need for validating translational relevance of mouse models with respect to neuroinflammation such that novel insights [10–14], therapeutic hypotheses, and targets are more likely to translate to clinical success.

Large-scale AD GWAS studies have provided genetic hypotheses for neuroinflammation in AD [15–23] and have consequently altered the perspective away from classical inflammatory pathways and toward innate immune dysfunction. Further support for a role of innate immune dysfunction in AD lies in recent transcriptomic characterization of AD brains which identified strong induction of an immune/microglia-related gene network in AD subjects [24]. Deeper characterization using directed probabilistic Bayesian networks identified the immune/microglia network as the module most strongly associated with the pathophysiology of Late-Onset Alzheimer’s disease (LOAD). This Bayesian network consists of five immunologic signaling families including the family of genes associated with complement signaling which included TYROBP, TREM2, and CD33 [23]. Loss of function mutations, specifically in TREM2 and TYROBP, linked with neurodegeneration originally via Nasu-Hakola disease and frontotemporal dementia were subsequently confirmed by several GWAS studies [25–28]. TREM2 and CD33, both extracellular microglial-enriched receptors associated with innate immune signaling, are known drivers of human AD pathogenesis [16–18, 29]. Follow-up functional studies of TREM2 and CD33 biology provide converging data demonstrating that defects in Aβ clearance may underlie the microglial-associated risk of AD pathophysiology (reviewed in [22]).

TREM2 and CD33 expressing microglia are known to localize around Aβ plaques in AD [30–32], and as such, we utilized laser capture microdissection to focus our current molecular profiling on the Aβ pathological niche. We provide a contextual view of tissue gene expression in a mouse model of AD with confirmed overlap with human AD-associated signatures. We hypothesize that beyond the enrichment of causal human AD genes, there exists additional genetically associated biology relevant to the development of AD pathology. Accordingly, data regarding alterations in gene expression around areas of plaque deposition could inform on local networks for potential therapeutic intervention as well as improve our understanding of local Aβ-related changes to enable better model systems to validate hypotheses in future studies.

## Methods

### Animals

Male transgenic (Tg) mice expressing APP KM670/671NL (Swe) and APP V717F (TgCRND8) and age-matched wild-type (WT) littermates were used in whole cortex and laser capture RNAseq evaluations as well as in situ hybridization studies. Tg female mice carrying the APPSwe mutation (Tg2576) [33] and age-matched WT littermates were used for the whole-cortex evaluations. Mixed sex cohorts were not available, precluding any evaluation of sex differences in gene expression. Mice were bred for Merck at Taconic Farms (Hudson, NY, USA) and single housed in an Association for Assessment and Accreditation of Laboratory Animal Care accredited facility that was maintained on a 12-h light/dark cycle (lights on at 0700 h). Food and water were available ad libitum. Principles of laboratory animal care were followed, and all studies were previously approved by the Institutional Animal Care and Use Committee and were performed in accordance to the Guide for the Care and Use of Laboratory Animals as adopted and promulgated by the National Institutes of Health (Library of Congress Control Number 2010940400, revised 2011).

### Whole cortex RNA extraction and RNA sequencing

For whole cortex analysis of gene expression, TgCRND8 mice were terminally sacrificed using CO_2_ at 1.5 (*n* = 4), 3 (*n* = 6), 4.5 (*n* = 3) 6 (*n* = 5), or 10 months (*n* = 7) of age. Corresponding WT littermates were also sacrificed at 1.5 (*n* = 6), 3 (*n* = 6), 4.5 (*n* = 4) 6 (*n* = 5), or 10 months (*n* = 6) of age. Tg2576 mice and corresponding WT controls were sacrificed similarly at 3, 6, 9, 12, and 15 months of age (*n* = 4 per group/age). After decapitation, the cortex was removed and rapidly frozen in liquid nitrogen and stored at − 80 until processing. RNA was extracted from TgCRND8 and corresponding control animals using Qiagen RNeasy Mini kit (Qiagen). The Magmax 96 for Microarray RNA extraction kit (Life Technologies) was used to for Tg2576 and corresponding control animals. RNA quality in all samples was assessed with the Agilent2100 Bioanalyzer. Libraries for RNA sequencing were prepared by Beijing Genomics Institute (BGI, Philadelphia, PA) using 100 ng of purified RNA and the Truseq stranded total RNA RiboZero library preparation kit (Illumina) strictly following the Illumina guide (15031048 E?). Briefly, rRNA was depleted with rRNA Removal Mix (RRM) and then fragmented into ~ 160 bp fragments. rRNA-depleted RNA fragments served as templates for first-strand cDNA synthesis using random hexamer-primers, followed by second stand synthesis with the addition of buffer, dNTPs, RNase H, and DNA polymerase I. Double-stranded cDNA was purified using the QiaQuick PCR extraction kit (Qiagen) followed by end repair, base A addition, and ligation of sequencing adapters. Ligated fragments were purified by magnetic beads and amplified via PCR. The resulting library products quantified with the Agilent2100 bioanalyzer and were sequenced using an Illumina HiSeqTM 4000 for a total of 3GB of 50 bp paired-end read data per sample.

### Thio-S stain and plaque laser capture

Fresh frozen TgCRND8 brains (*n* = 12; 6 months old) were sectioned axially (12 μm) and freeze mounted onto PEN-membrane slides (Applied Biosystems). Slides were kept on dry ice until fixation in 75% ethanol for 30 min at room temperature followed by 5 min incubation in a 1% Thioflavin-S solution (Sigma). Sections were then dehydrated in 70% ethanol for 5 min, 90% ethanol for 2 min, and 100% ethanol for 2 min. Slides were air-dried and stored at room temperature (RT) in a desiccator until LCM. Sections were cut on the laser capture system within 24 h of staining. An Arcturus laser capture system was used to gather plaque areas (defined as tissue area extending ~ 100 μm beyond the perimeter of the Thio-S positive plaque) and non-plaque areas from TgCRND8 tissue sections. For each TgCRND8 sample, 127 ± 50 plaque areas were isolated, lifted onto Arcturus caps, and processed according to manufacturer’s directions using a PicoPure Arcturus RNA extraction kit (Applied Biosystems). Non-plaque areas were gathered from the remaining Thio-S negative tissue parenchyma.

### In situ hybridization/immunohistochemistry co-labeling

TgCRND8 (*n* = 4, 35 weeks old) brains were drop fixed in 10% buffered formalin for 24 h at RT prior to processing and embedding in paraffin. Coronal sections (5 μm thick) were collected on a rotary microtome and mounted onto superfrost™ plus slides. Trem2 and CD33 messenger ribonucleic acid (mRNA) were labeled in the formalin-fixed paraffin-embedded TgCRND8 sections using RNAscope VS® (Advanced Cell Diagnostics, Inc., Hayward, CA) technology. Sections were loaded onto a Discovery Ultra® (Ventana Medical Systems, Tucson, AZ) immunostainer, where slides were deparaffinized and treated with protease according to manufacturer’s recommendations. Trem2- or CD33-specific mRNA target probes were provided by the manufacturer (Trem2: gene ID 83433 targeted bps 2 through 1081 of the complementary deoxyribonucleic acid (cDNA) sequence, CD33: gene ID 12489 targeted 448 through 1408 of the cDNA sequence). Following signal amplification, target mRNA was visualized with 3,3′-diaminobenzidine (DAB) substrate. A hydrogen peroxide quenching step was performed to prepare the samples for β-amyloid co-labeling. An anti-β-amyloid antibody (6E10, Biolegend) was diluted in 10% goat serum to a working concentration of 0.1 μg/ml to label amyloid plaques. Following incubation of the primary antibody, Discovery Omnimap-Mouse HRP® secondary antibody was applied to the sections. Ventana’s Discovery Purple Kit® was used to contrast the brown ISH signal. After staining, sections were counterstained with hematoxylin. Sections were scanned with an Aperio XT slide scanner (Leica Biosystems) at × 40 magnification.

### NanoString RNA counting methods

NanoString nCounter technology (http://www.nanostring.com/) allows expression analysis of multiple genes from a single sample. We performed nCounter multiplexed target profiling of 400 custom transcripts. The nCounter codesets were selected prior to the RNAseq data; however, significant representation of differentially expressed genes in the whole cortex and LCM datasets were captured by the custom chips. Fifty nanograms of total RNA per sample was used in all described nCounter analyses according to the manufacturer’s suggested protocol. Raw counts for key targets are illustrated in Additional file 1: Figure S1 confirming the RNAseq data via an alternative gene expression profiling platform. Additionally, in Additional file 2: Figure S2, the fold-change data for each transcript on the custom NanoString panel is plotted versus the RNAseq data, for both the whole cortex samples and LCM samples.

### RNA sequencing of LCM samples

Purified RNA (1 ng/sample) from 24 LCM samples (12 transgenic plaque region and 12 transgenic distal non-plaque region) were used for cDNA production using a SMARTer Ultra Low RNA Kit (Clontech, Mountain View, CA) following the manufacturer’s instructions with minor modifications. After cDNA amplification and purification, half of the resulting cDNA was used as input for the Nextera XT DNA Sample Preparation Kit (Illumina, San Diego, CA) using a Nextera XT Index Kit (Illumina). Nextera XT sample preparation uses an engineered transposome to fragment and attach sequencing adapters to DNA samples. Illumina Nextera XT protocols were followed for library preparation with 1.0 volumes of purification Agentcourt Ampure XP beads (Beckman Coulter, Beverly MA). No normalization was performed on individual libraries. The library quality was determined using a NanoDrop 1000 (Thermo Scientific, West Palm Beach, FL) to measure library concentration and Bioanalyzer 2100 (Agilent Technologies, Santa Clara, CA) with a DNA 1000 chip to assess library size and quality. Libraries were pooled, and sequencing was performed using an Illumina HiSeq 2500 (Illumina) sequencer with Rapid paired-end sequencing of 76 × 76 bases. Fastq files were generated and demultiplexed using the bcl2fastq version 1.8.4 program.

### Data processing

Alignment and differential gene expression analysis was performed in Omicsoft Array Studio version 7.2.2.29. Briefly, cleaned reads were aligned to the human B37.3 genome reference using the Omicsoft Aligner with a maximum of four allowed mismatches. Gene level counts were determined by the RSEM algorithm as implemented in Omicsoft Array Studio and using RefGene transcript annotation prepared on March 21, 2014. Resultant FPKM data was subsequently ratioed to the average of the WT plaque samples for each gene prior to analysis. Only genes with ≥ 10 counts in 50% of the samples in at least one treatment group where considered detected and included in analysis.

### Statistical analysis

Differentially expressed genes were identified by Pearson correlation or *t* test using MatLab R2010b (Mathworks). A *p* value cutoff of < 0.001 was used to identify differentially expressed genes. The FDR corresponding to this *p* value is given in each of the comparisons to convey relative signature confidence. Set annotation analysis was performed by comparing input sets to GeneGo (www.genego.com), Ingenuity (www.ingenuity.com), and KEGG (www.genome.jp/kegg/) pathway sets. Bonferroni-corrected hypergeometric *p* values (expectation (*e*) values) of less than 0.1 were considered significant overlap between sets. Heatmaps were generated in MatLab R2010b using agglomerative clustering.

## Results

### Age- and strain-related transcriptomic changes in AD models

**Fig. 1:**
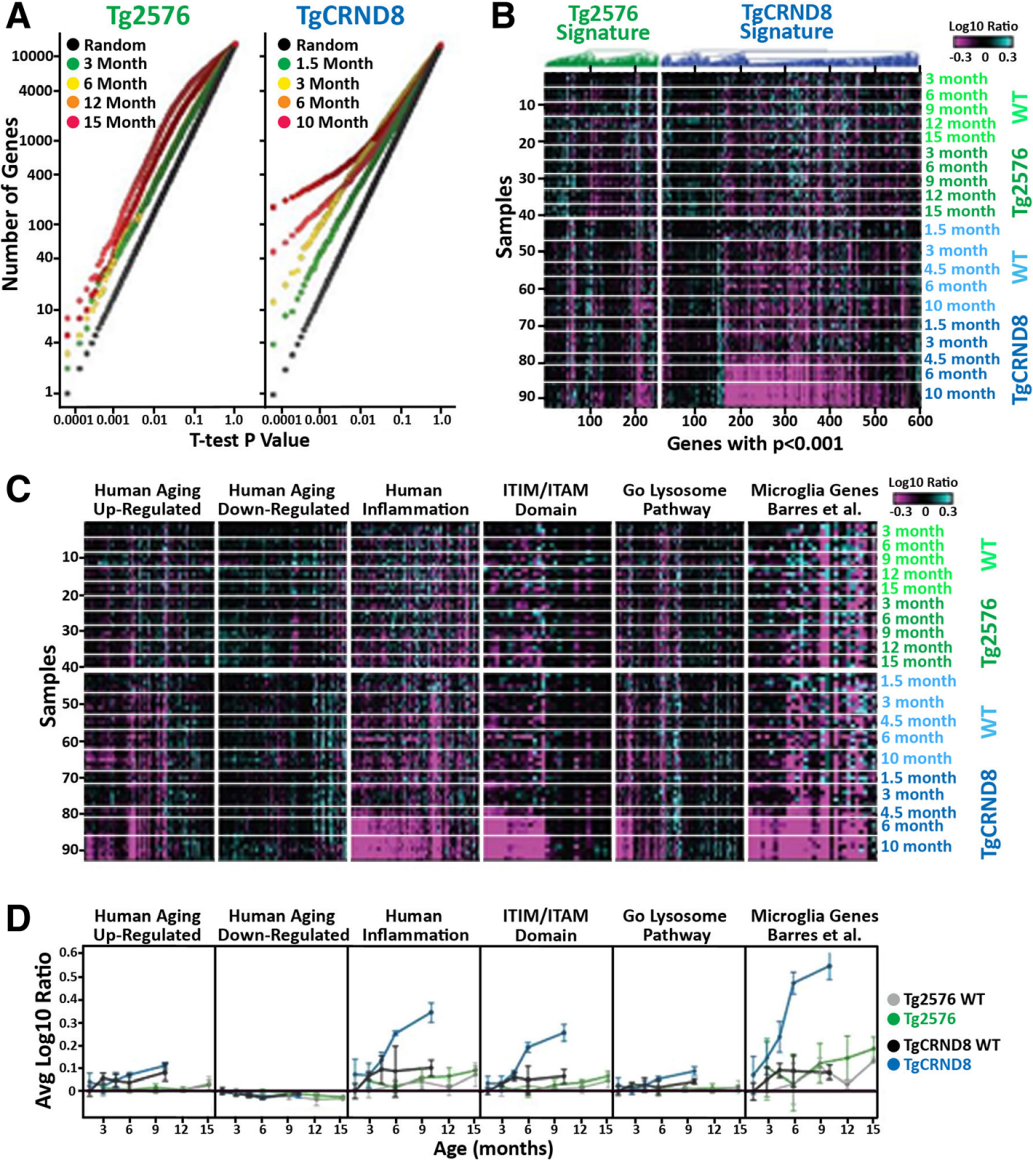
Transcriptional changes in aging Tg2576 and TgCRND8 brain. **a** Student’s *t* test *p* value distributions for gene expression differences between WT and Tg2576 (left plot) or TgCRND8 (right plot) at different ages. Gray line indicates expected false discovery rate (FDR) given multiple test comparisons. **b** Heatmap showing log_10_ ratio values from each sample (*y*-axis) for each gene (*x*-axis) with *t* test *p < 0.001* between Tg2576 (green) and TgCRND8 (blue) versus WT littermate controls at one or more ages. Samples are ordered manually by genotype as indicated. Genes are ordered by test and agglomerative clustering. **c** Heatmaps showing log_10_ ratio values from each sample (*y*-axis) for each gene (*x*-axis) within the indicated gene sets. Samples are ordered manually by genotype as indicated. Genes are ordered by agglomerative clustering within each set. **d** Signature scores (average of gene values in C ± standard deviation) for the indicated gene sets over age in Tg2576 (green) and WT littermate control (gray) as well as TgCRND8 (blue) and WT littermate controls (black)

First, we carried out RNAseq on cortexes isolated from Tg2576, TgCRND8, and WT controls. Data revealed a genotype-dependent increase in gene expression differences with respect to age primarily in TgCRND8 animals (Additional file 3: Table S1). In WT animals, relatively few genes showed age-dependent changes. Only 28 genes had Pearson correlation coefficient (|ρ|) > 0.75 with respect to age in 3–15 month Tg2576 littermate controls, while just 112 genes correlated similarly with age in 1.5–10 month TgCRND8 littermate controls. Similarly, 42 genes had |ρ| > 0.75 with age in 3–15 month Tg2576 animals. In contrast, 1994 genes had correlation |ρ| > 0.75 with age in 1.5–10 month TgCRND8 animals. In order to focus on genotype-related differences without confounding normal aging signatures, we compared the expression differences in Tg2576 and TgCRND8 animals to their respective age-matched WT controls. A small number of genes were differentially expressed between Tg2576 and WT at all ages examined (Fig. 1a, b). Specifically, 43, 42, 86, 11, and 58 out of 15,857 detected genes were significantly different (*p < 0.001*) between WT and Tg2576 animals at 3, 6, 9, 12, and 15 months, respectively (FDR < 37, 38, 18, 100, and 27%, respectively). Combined, 235 genes displayed a significant difference (*p < 0.001*) between Tg2576 and WT at one or more ages tested, 125 genes (53%) of which were upregulated. However, a strong progression of genotype-related expression changes was observed in TgCRND8 animals (Fig. 1a, b). At 1.5 months of age, 52 genes had significantly different expression in the TgCRND8 cortex compared to WT controls (*p < 0.001*, FDR < 29%); by 3 months of age, 108 genes (FDR < 14%) were similarly regulated and this number increased to 170 (FDR < 9%) and 351 (FDR < 4%) at 6 and 10 months, respectively. Combined, 582 genes displayed a significant difference (*p < 0.001*) between TgCRND8 and WT at one or more ages tested, 464 (80%) of which were upregulated. Numerous gene pathways were significantly (Bonferroni-corrected *p* value (*e*) < 0.1) enriched among the genes upregulated in TgCRND8 cortex, predominantly those associated with immunity inflammation lipid metabolism and protein salvage (Additional file 4: Table S2). For example, numerous lysosomal and ITIM (immunoreceptor tyrosine-based inhibition motif; S/I/V/LxYxxI/V/L) and ITAMs (immunoreceptor tyrosine-based activation motif; consensus sequence YxxI/Lx6-12YxxI/L) signaling genes exhibited age-dependent increases in expression in TgCRND8 mice (Fig. 1c, d). However, no biological pathways were identified as significantly enriched among the smaller number of genes downregulated in Tg2576 or in either of the upregulated or downregulated gene sets in Tg2576 despite Tg2576 mice presenting with plaque pathology (Additional file 5: Figure S3).

### Translational transcriptomic profiling

Next, we assessed whether orthologs of the previously identified human cortical gene networks impacted with aging and AD were modulated with age in the mouse models examined (Additional file 6: Table S3) [34]. As expected, little progression of the human aging signatures was observed in mouse cortex across the ages examined (Fig. 1c, d). Although some of the aging signature genes changed significantly, the trends across all genes with age and the differences between Tg2576/TgCRND8 and WT controls were not significant (Fig. 1c, d). The human inflammatory signature was upregulated in aged specimens. This signature was robustly potentiated in AD subjects regardless of age and also exhibited strong progression with TgCRND8 aged animals. Indeed, this signature was highly enriched (*p < 3e−53*) among the 464 genes differentially upregulated (*p < 0.001*) between TgCRND8 and WT controls (Fig. 1). Similarly, we examined whether the brain cell-type specific gene sets identified by Zhang et al., 2013 were enriched among the up or down arms of the Tg2576 or TgCRND8 signatures. Significant overlap was only observed with the microglial gene set, which was highly enriched (*p < 6e−24*) among the genes upregulated in TgCRND8 (Fig. 1c, d) [23].

### Plaque transcriptomic signature

**Fig. 2:**
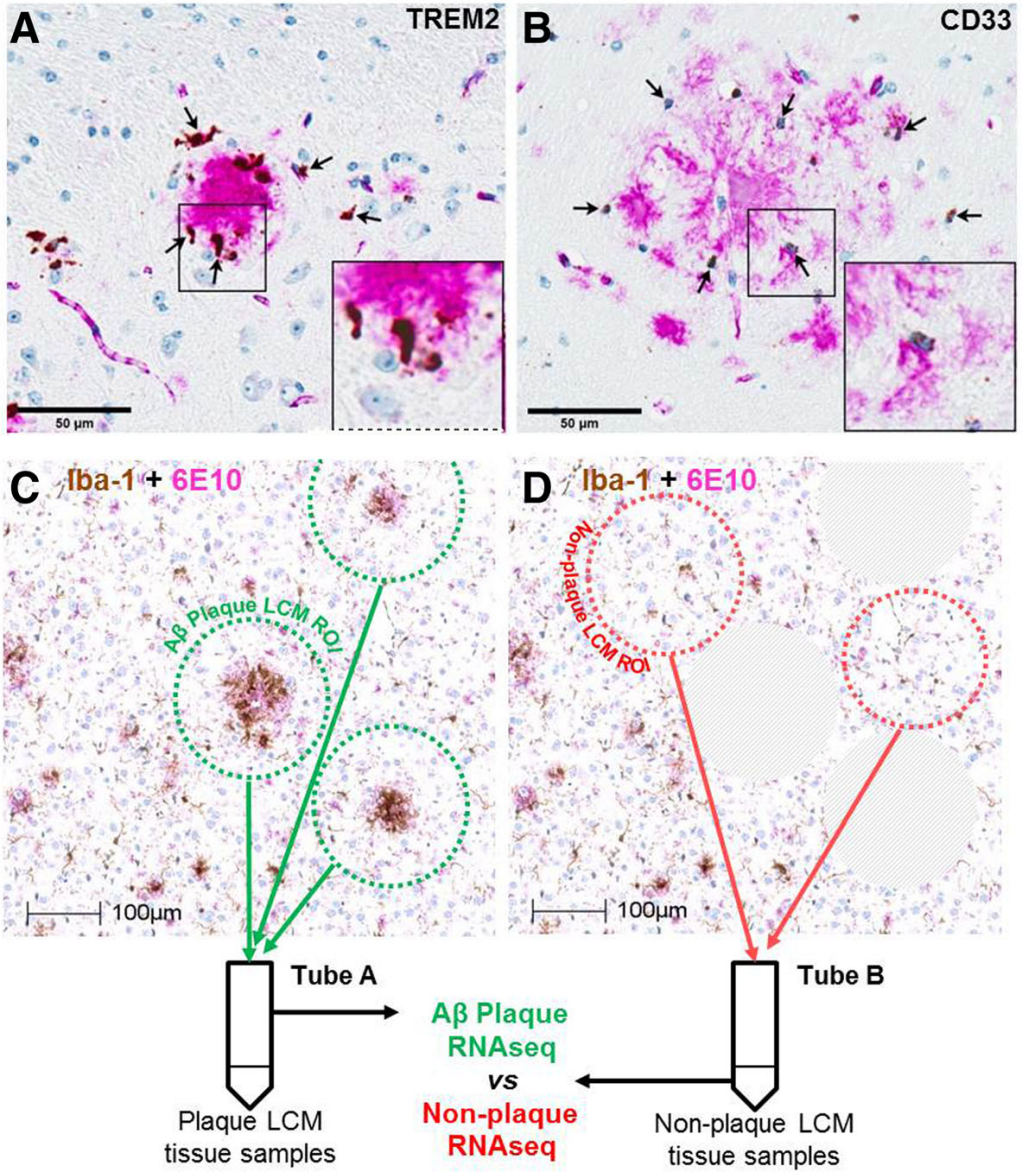
Localization of TREM2 and Cd33 around amyloid-beta (Aβ) plaques in the aged TgCRND8 mouse. Brain slices were prepared from 35-week-old wild-type and TgCRND8 mice and processed for amyloid-beta immunohistochemistry (6E10 labeling, magenta) combined with TREM2 (panel **a**) or CD33 (panel **b**) in situ hybridization via RNAScope (brown). Visualization of TREM2 and Cd33 confirms their associated expression pattern with Aβ pathology, supporting the rationale for laser capture microdissection of Thio-S-labeled plaques for transcriptome-wide RNA sequencing. Representative images show section sampling for LCM and RNA seq (**c**, **d**). Images show Iba1 (brown) and 6E10 (magenta) immunostainings. Scale bars, 50 and 100 μm

**Fig. 3:**
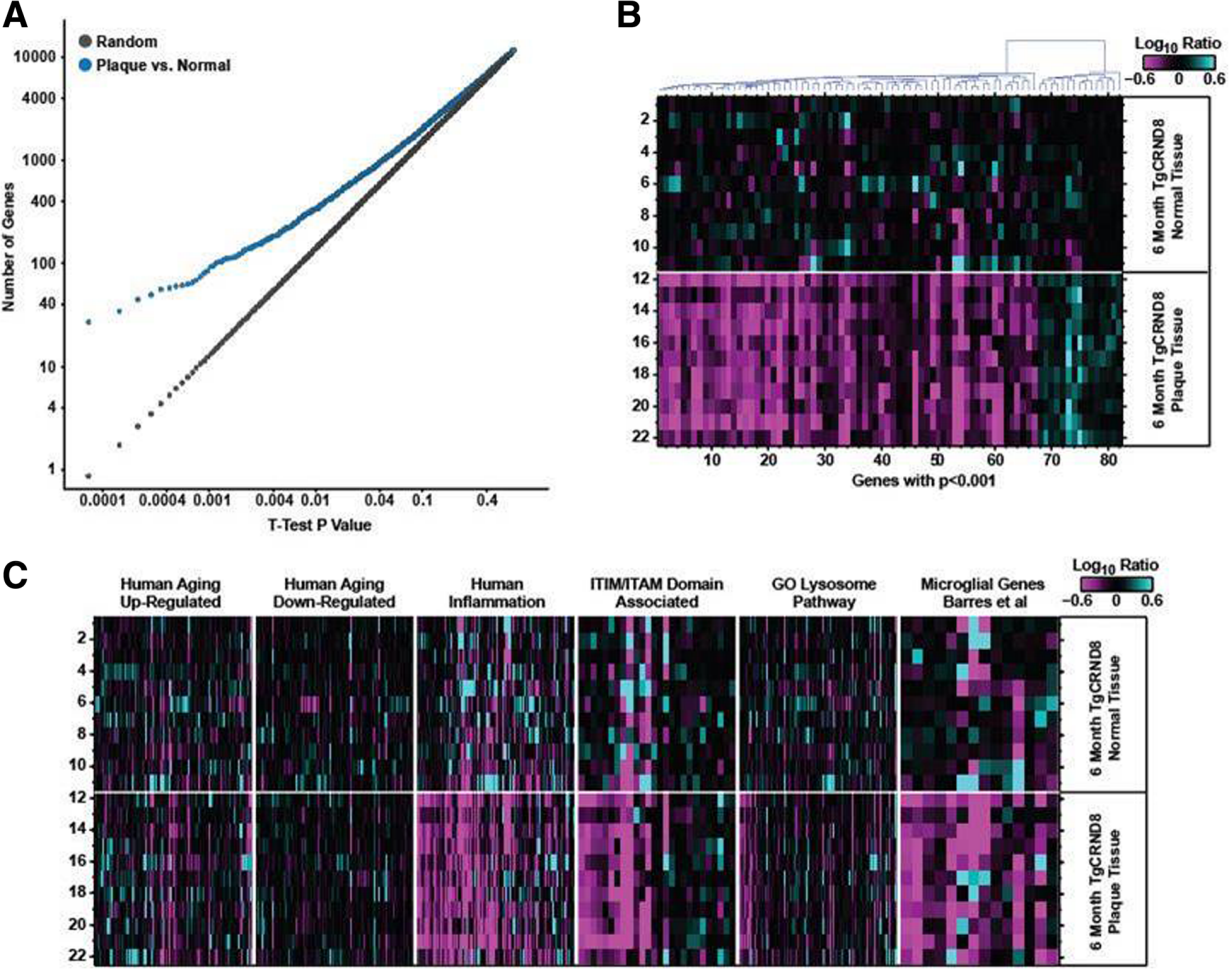
Plaque-niche gene expression differences in 6-month-old TgCRND8. **a** Student’s *t* test *p* value distributions for gene expression differences plaque and normal samples in TgCRND8 cortex at 6 months of age. Gray line indicates expected false discovery rate (FDR) given multiple test comparisons. **b** Heatmap showing log_10_ ratio values from each sample (*y*-axis) for each gene (*x*-axis) with *t* test *p* value < 0.001 between plaque and normal tissue in TgCRND8 cortex. Samples are ordered manually by genotype as indicated. Genes are ordered by agglomerative clustering. **c** Heatmaps showing log_10_ ratio values from each sample (*y*-axis) for each gene (*x*-axis) within the indicated gene sets. Samples are ordered manually by genotype as indicated. Genes are ordered by agglomerative clustering within each set

**Fig. 4:**
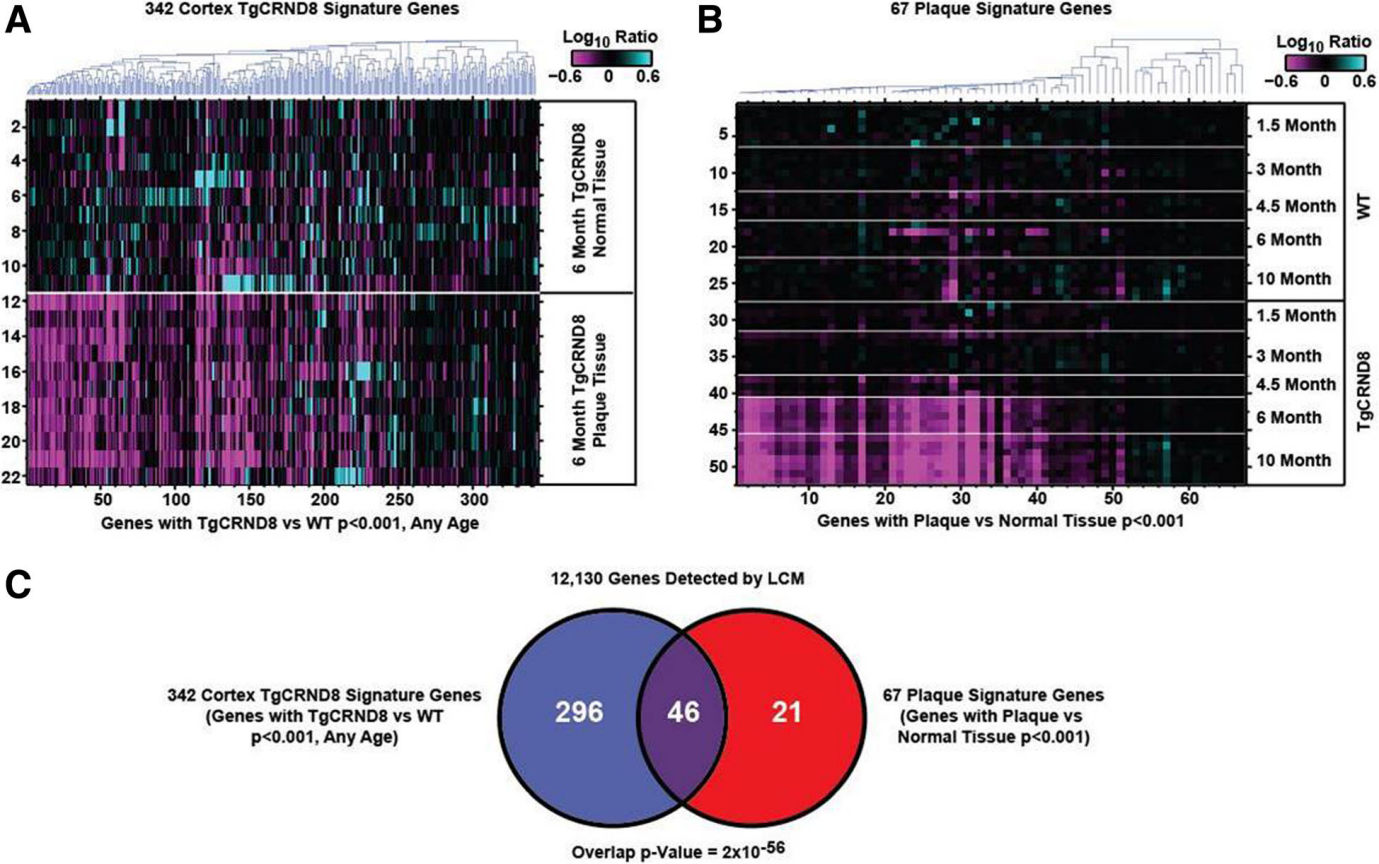
Overlap of TgCRND8 progression signature and plaque-niche signature. **a** Heatmap showing log_10_ ratio values from each LCM sample (*y*-axis) for each gene (*x*-axis) detected in both the LCM and whole cortex studies and with *t* test *p < 0.001* between TgCRND8 and WT cortex at one or more ages. Samples are ordered manually by genotype as indicated. Genes are ordered by agglomerative clustering. **b** Heatmap showing log_10_ ratio values from each whole cortex sample (*y*-axis) for each gene (*x*-axis) detected in both the whole cortex and LCM studies and with *t* test *p < 0.001* between plaque and normal tissue in TgCRND8 cortex. Samples are ordered manually by genotype as indicated. Genes are ordered by agglomerative clustering. **c** Venn diagram depicting number of genes in common between signatures in **a** and **b**, and the associated hypergeometric *p* value given 12,130 genes detected in both experiments

In order to establish that the identified gene networks, particularly the human-related disease signatures, were not only temporally but also regionally associated with Aβ pathology LCM was performed around Thio-S-labeled sites. We used the in situ hybridization pattern of TREM2 and CD33 around Aβ plaques in the aged TgCRND8 mouse (Fig. 2) to qualitatively define the radius of the laser capture parameters. Representative images were stained with Iba-1 and 6E10 (Fig. 2c, d) to illustrate plaque and non-plaque areas processed for RNA seq. Brain slices prepared from aged TgCRND8 mice and processed for Aβ immunohistochemistry (6E10 labeling, magenta) combined with TREM2 (panel A) or CD33 (panel B) in situ hybridization via RNAScope (brown) confirmed their associated expression pattern with Aβ pathology in the TgCRND8 model and supported our rationale for laser capture microdissection not just of Thio-S-labeled plaques (as performed by [35]) but also the surrounding penumbra (Fig. 2). RNAseq of laser capture samples revealed transcriptomic differences between plaque niche and non-plaque TgCRND8 tissue. In TgCRND8 mice, 82 out of 12,461 detected genes were significantly (*p < 0.001*; FDR < 15%) regulated compared to non-plaque TgCRND8 tissue; 67 of these genes were upregulated and 15 were downregulated (Fig. 3a, b; Additional file 7: Table S4). Numerous gene pathways were significantly (*e* < 0.1) enriched among the genes upregulated in the plaque niche, predominantly those associated with vesicles, immunity, inflammation, and protein salvage, for example lysosomal or immunoreceptor tyrosine-based activation/inhibition motif (ITIM/ITAM) signaling genes (Fig. 3c, Additional file 4: Table S2). As in the TgCRND8 versus WT comparison, the human inflammation signature (*p < 3e-34*) as well as the microglial-specific signature (*p < 7e-7*) were significantly enriched among the genes upregulated within the plaque niche signature, while the other human and cell-type gene sets were not (Fig. 3c). The expression differences in TgCRND8 mice and those enriched in the plaque niche were highly overlapping. The majority of the genes upregulated in TgCRND8 cortex compared to WT animals were also expressed higher in CRND8 plaque versus non-plaque (Fig. 3a). Conversely, the majority of the genes upregulated in the plaque versus non-plaque comparison were also upregulated in 6–10-month-old TgCRND8 animals compare to WT (Fig. 4b). Several genes (12,130) were detected in both experiments, including 342 genes that were upregulated in TgCRND8 vs WT cortex (*p < 0.001* at any age) and 67 genes upregulated in plaque vs non-plaque (*p < 0.001*), of which 46 were overlapping between both signature sets (*p < 2e-56*) (Fig. 4c). We confirmed the findings from our RNAseq data using a customized NanoString nChip showing that key plaque-associated genes were upregulated (*Trem2, Tyrobp, Cd68, Clec7a, Tspo, Itgfax*) (Additional file 1: Figure S1). Data were analyzed for expression of a set of markers associated with resident microglia or peripheral macrophages as outlined by Hickman et al. to characterize the phenotype of cells surrounding the plaque [36]. Results show that 7 out of the top 25 most abundant genes in resident microglia were significantly upregulated in the plaque niche compared to non-plaque samples (Additional file 8: Figure S4). Conversely, only 1 gene associated with peripheral macrophage expression was significantly upregulated in the plaque niche, *Complement C4-b* (*C4b)* (Additional file 8: Figure S4). This phenotypic analysis was more pronounced in whole cortex samples resulting in 13 out of the top 25 microglial-specific genes were significantly upregulated in TgCRND8 mice compared to WT compared to only 1 gene in the peripheral macrophage signature (Additional file 8: Figure S4).

**Fig. 5:**
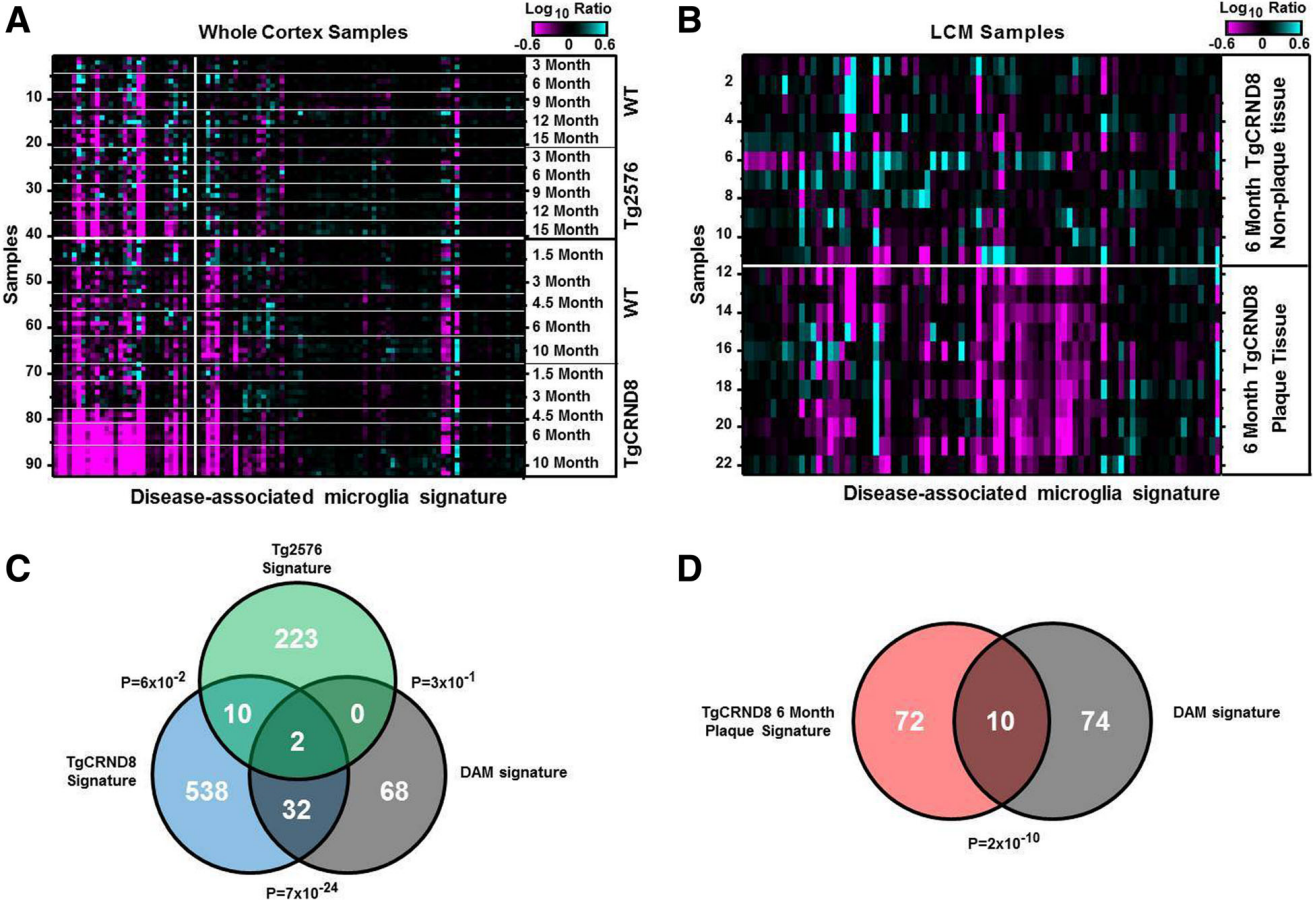
Overlap of TgCRND8 progression signature and plaque-niche signature. **a**, **b** Heatmap showing log_10_ ratio values from each whole cortex sample (**a**) or each LCM sample (**b**) for genes reported to be expressed higher (> 1.5 fold, *p < 1e-5*) in the DAM population [37]. Samples are ordered manually by genotype and age as indicated. Genes are ordered by agglomerative clustering. **c** Venn diagram depicting number of genes in common between the Tg576/TgCRND8 whole cortex signatures (*p < 0.001*, any age) and the genes expressed higher (> 1.5 fold, *p < 1e-5*) in the DAM cells. **d** Venn diagram depicting number of genes in common between the TgCRND8 LCM plaque signature (*p < 0.001*) and the genes expressed higher (> 1.5 fold, *p < 1e-5*) in the DAM population

To further characterize the microglia phenotype observed in the TgCRND8 plaque niche, we compared the gene signatures identified here to those of the microglial subtype, disease-associated microglia (DAM), recently shown to surround plaques in another mouse AD model expressing five familiar AD mutations (5XFAD) [37]. Indeed, genes reported to be expressed higher in the DAM population (> 1.5 fold, *p < 1e-5*) were strongly enriched among the upregulated gene signatures in both the TgCRND8 whole cortex and the LCM-dissected TgCRND8 plaque tissue signatures (*p = 4e-25* and *p = 8e-13*, respectively) (Fig. 5a–d; Additional file 4: Table S2, Additional file 6: Table S3, and Additional file 7: Table S4). Across all three gene sets, eight genes were commonly expressed: *Ccl6, Cd9, Ctsz, Gusb, Lyr2, Npc2, Trem2,* and *Tyrobp*. In contrast, the DAM marker genes were not significantly represented among the Tg2576-related signatures (*p = 0.3*) (Fig. 5a, c). In additional analyses, expression of markers of M1 and M2 microglia as outlined in [38, 39] were also analyzed for differential expression in plaque vs. non-plaque samples as well as in whole cortex samples from TgCRND8 compared to WT controls. Results show a significant increase in expression of only one marker of M1 activation, *CD16a*, in the plaque niche compared to non-plaque controls (*3.06-fold*; *p < 0.0001)*. No markers of M2 activation were significantly upregulated in plaque samples compared to non-plaque. Whole cortex TgCRND8 displayed significant (*p < 0.001*) expression of four markers of M1 activation, *CXCL10 (34.52-fold*; *p < 0.0001)*, *CCR5 (1.51-fold*; *p < 0.0001)*, *CD86 (2.25-fold*; *p < 0.0001*), and *CD16a (2.62-fold*; *p < 0.0001)* in TgCNRD8 compared to WT, and only two markers of M2 activation/repair, *Clec7a (72.33-fold*; *p < 0.0001)*, and *TGFβ* (*2.23-fold*; *p < 0.0001*).

## Discussion

Many studies have demonstrated a strong association between inflammation, innate immunity, and amyloid plaques in both human AD and preclinical models [23, 40]. Data described here extend and deepen prior characterizations of two different APP transgenic mouse models of AD, the Tg2576 and TgCRND8 lines, by leveraging next-generation RNA sequencing to enable comprehensive and progressive transcriptional profiling of cortical Aβ plaque pathology. Data also provide context for microglial reactivity in the plaque niche; the majority of transcriptional changes observed in aging TgCRND8 cortical brain homogenates were specifically enriched within the plaque niche compared to samples from non-plaque regions of the cortex from TgCRND8 mice. These data provide crucial context for translational relevance of two mouse models of AD and insights into transcriptional regulation in the plaque niche.

The Tg2576 mouse overexpresses a variant form of APP harboring dual Swedish mutations (KM670/671NL) under the control of the hamster PrP promoter, resulting in substitutions of two amino acids, lysine (K) and methionine (M) to asparagine (N) and leucine (L) [41–43]. In vitro, this mutation results in elevated production and secretion of both Aβ40 and Aβ42, as this mutation lies immediately adjacent to the β-secretase site on APP resulting in higher affinity to BACE1 compared to wild-type APP [43]. The Swedish mutation does not generally lead to amyloidosis by shifting the ratio of Aβ42/Aβ40. The Swedish mutation predominantly leads to the secretion of Aβ40, a less amyloidogenic peptide. Similarly, the TgCRND8 mouse also overexpresses the Swedish APP mutation under control of the hamster PrP promoter but additionally contains the Indiana (V717F) mutation in the human APP transgene. In contrast to AD-associated residues modulating β-secretase-mediated cleavage of APP, pathogenic mutations such as V717F occurring at the ϒ-secretase processing site of APP increase the ratio of Aβ42/Aβ40, lead to elevated secretion of the intrinsically aggregation prone Aβ42, and increase the ratio of Aβ42/Aβ40. It is widely accepted that Aβ42 is the major component of amyloid plaques in AD brain, despite observations that the concentration of Aβ40 is significantly higher than Aβ42. In vitro, Aβ40 and Aβ42 are capable of forming interlaced fibrils [44]; however, the rate of aggregation is controlled in part by the ratio of Aβ40 with Aβ42, as peptide mixtures are less amyloidogenic than Aβ42 alone [45–49]. In line with this view, transgenic mice expressing solely Aβ40 do not develop amyloid pathology or form insoluble aggregates [50]. Similarly aligned, the Tg2576 model exhibits slower Aβ pathology compared to TgCRND8 mice, with the extent of insoluble Aβ levels being more severe in TgCRND8. Here, we exploit these fundamental differences between two APP models to broadly evaluate how that impacts secondary responses to Aβ plaque burden by whole transcriptome sequencing.

Our findings show that the human AD neuroimmune signature develops progressively in the TgCRND8 mouse cortex, coincident with known trajectories of Aβ deposition, but not in the Tg2576 mouse, raising intriguing questions about how the brain responds to different composition states of Aβ. The signature changes observed in brain homogenates highly overlap with the local expression profile of the plaque niche. One possibility is the degree of insoluble Aβ aggregate burden directly triggers the transcriptional changes observed in the TgCRND8. However, it is more likely that a constellation of contributing neurodegenerative triggers occur within the plaque-laden niche. For example, alterations in synaptic density, the extracellular matrix proteome (i.e., matrisome), the local secretome, and possibly changes in lipid composition may vary with specific Aβ plaque pathology [48]. Future in vitro studies exploring whether exposure to monomeric, oligomeric, and fibrillar Aβ species which are sufficient to induce the observed transcriptional responses would help to address the specificity and complexity of the sequencing changes observed here via laser capture of Aβ plaques.

A similar study utilizing LCM to isolate protein-rich amyloid plaques in the absence of surrounding cellular penumbra from two clinical AD cases previously reported an enrichment of glial fibrillary acidic protein (GFAP), a marker for reactive astrocytes, and vimentin, an intermediate filament, in Thio-S positive plaques in AD patients [35]. In this proteomic study, several subunits of lysosomal ATPase were concentrated in the plaque, along with other markers of the ubiquitin-proteasome system, collectively indicating an activation of protein degradation mechanisms. Liao et al. also identified many heat shock proteins, specifically HSP90, enriched in the plaques [35]. Our current RNAseq data also demonstrate a significant (*p < 0.001*) increase of nearly 3-fold in the expression of GFAP in the plaque niche compared to non-plaque tissue. While reactive astrocytes have been shown to express high levels of heat shock proteins, we did not observe changes in heat shock family transcripts despite evidence of enriching astrocyte-specific markers in the plaque niche of the TgCNRD8 mouse. However, consistent with human proteomic analysis, the current transcriptomic insights presented here (Additional file 4: Table S2) confirm a significant upregulation in the expression of genes associated with lysosomal function in plaque areas compared to non-plaque. The observations of alterations in lysosomal pathways support the notion that the plaque core and its surrounding glia comprise a hot spot for autophagy-lysosomal activity and that pathways related to protein homeostasis and phagocytosis of extracellular amyloid plaques or other neurodegenerative debris are altered in the plaque niche.

Previous histological studies unequivocally validated a discrete regional association between Aβ plaques and microglia in human and mouse models of AD, and current data reveals a complex picture regarding the upregulation of markers of microglia, potentially indicating a phenotypic difference between plaque-associated microglia and non-plaque-associated microglia [51–57]. Not surprisingly, canonical markers of microglia such as fractalkine (*cx3cr1*), *cd68*, and *itgam* (CD11b) were significantly upregulated in the plaque niche compared to non-plaque areas: 2.68, 3.0, and 2.0-fold, respectively. Significant increases in the expression of *cx3cr1* and *cd68* were also sufficiently robust to detect in whole cortex analysis in 6-month-old animals (*p < 0.001*); in contrast, CD11b was not significantly upregulated in the whole cortex at this age. Interestingly, the classical marker of activated microglia *aif1* (Iba1) demonstrated no significant change between plaque-associated samples and non-plaque tissue. However, the longitudinal profiling suggests that *AIF1* induction emerges as a later adaptation to amyloid plaques, as it is more highly upregulated in the 10-month-old TgCRND8 cohort (2.1-fold) compared to 6-month TgCRND8 animals (1.5-fold), the age at which the plaque niche was profiled in this laser capture study. These observations serve to highlight that classical markers of microglia phenotypic state carry unique time-courses and regional-specificity which should be accounted for when utilizing a single or limited subset of cell-specific markers to draw inferences on neuroimmune status, responses to treatment interventions, and consequent interpretations on efficacy of interventions.

Another common method to evaluate microglial states has been through more extensive marker classifications adopted from peripheral macrophage profiling in order to draw conclusions regarding the M1 vs M2 polarization status of resident CNS microglia [58]. According to this paradigm, M1 microglia are associated with the production of pro-inflammatory cytokines such as tumor necrosis factor-α (*TNFα*), interleukin-1β (*IL-1β*), and interleukin-6 (*IL-6*), as well as the catalysis of nitric oxide (*NO*) from arginine and release of various state-specific chemokines. Alternatively, markers of the M2 activation and repair state include *YM1* (*Chi3l3*), non-TLR pattern recognition receptors such as dectin-1 (*Clec7a*), *CD200R*, and *MRS1*, along with *Arg1* which favors arginine metabolism, and ALOX12 and 15 involved in arachidonic acid metabolism. In the current study, we did not observe a clear distinction of the M1 versus M2 phenotypes of microglia across broader panels; either in whole tissue comparisons to WT mice or focally around the Aβ plaque niche within the TgCRND8 mouse. Microglial functions are incredibly diverse and comprise a broad spectrum of adaptations from inflammation, neuroprotection, and neuroimmune processes including phagocytosis, clearance of cellular debris, expression of growth factors, production and release of cytokines, and synaptic pruning [59]. Possible explanations for a failure to see clear activation of an M1 or M2 phenotype include extreme heterogeneity of microglial phenotypes, the stage of TgCRND8 plaque progression in the current study, or the relative absence of other cardinal features of AD such as tau pathology or robust neuronal loss.

In recent years, microglial research has shifted away from the M1/M2 nomenclature, with the aid of tools such as specific microglia antibody-labeling and single-cell transcriptomics and proteomics analysis. Using these tools, research groups have been able to identify the microglial transcriptome in neurodegenerative states that are more diverse than M1/M2 polarization which is more phenotypic relevant. In one such study, using an AD model, disease-associated microglia or “DAM” cells surround the plaques and exhibit distinct genetic profiles that differ from homeostatic/non-disease-associated microglia [37]. We carried out comparative analyses between published DAM signature profiles and our data sets which revealed that DAM-signatures are present in the plaque-associated tissue in the TgCRND8 mice and this also correlates with age. From this gene set, 32 genes overlapped between TgCRND8 plaque niche versus DAM genes, with two of these genes being *Tyrobp* and *Trem2*. There were no genes overlapping in Tg2576 versus the DAM signature (Fig. 5c). Thus, our data suggests that the TgCRND8 model is a more relevant model to understand polarization states and plaque niche.

The TgCRND8 transcriptional profiles align with previous findings of robust transcriptional responses in recently discovered genetic links between microglia, innate immunity, and AD. Numerous GWAS and meta-analyses in various AD cohorts’ studies have identified novel microglial-associated receptors TREM2 and CD33 for AD [16, 18, 29]. This has naturally led to investigations of their roles in AD in preclinical models. In the current study, regional assessment of the Aβ plaque niche revealed TREM2 and its signaling adaptor TYROBP (which encodes the DAP12 protein) are significantly *(p < 0.001*) upregulated in the Aβ plaque niche compared to non-plaque areas. Both markers show a 3-fold increase in expression near the plaque compared to non-plaque samples. An expanded view beyond these hub genes confirms that several ITIM/ITAM-associated transcripts are also enriched around plaques. In TgCRND8 cortical homogenates, this innate immune panel was shown to be upregulated at 6 and 10 months of age, as well. It is important to highlight, however, that the ITIM/ITAM-associated module is not invariably associated with Aβ plaque pathology. In the Tg2576 model, we did not observe significant induction of this AD-implicated innate immune signature. Therefore, when juxtaposed with robust progressive signature induction in the TgCNRD8 mice, it is clear that not all mouse APP models are equivalent in this regard.

Exploring more closely specific ITIM/ITAM-associated changes, we identified novel dysregulated pathway nodes as potential modulatory factors, including genes known to be enriched in microglia such as receptor and non-receptor tyrosine kinases, as well as associated phosphatases predicted to inhibit phagocytic signal transduction. For instance, it is widely known that DAP12 signals via SYK induction and its downstream signaling may be negatively regulated by various phosphatases such as PTPN6 and INPP5D. Interestingly, not only do we observe robust SYK upregulation around amyloid plaques (> 3-fold) but associated protein tyrosine phosphatases PTPN6 and INPP5D are also enriched (upregulated 6.0-fold and 2.7-fold respectively). Both PTPN6 and INPP5D are involved in the signaling via inhibitory receptors found in innate immune cells, such as CD33 for example. Moreover, in a meta-analysis of major AD GWAS, the rs35349669 locus on chromosome 2 encoding INPP5D was recently identified as a novel AD risk-associated locus [60–62]. Within the plaque niche, we also observed that several inhibitory ITIM-containing receptors in addition to CD33 are also enriched, such as *Slamf9* (7.8-fold, *p = 0.011*), *FCGR1* (2.2-fold, *p = 0.002*), and *LAIR1* (2.0-fold, *p = 0.001*). Likewise, we identified several additional ITAM-associated receptors such as *FCGR*1 (2.2-fold, *p = 0.002*), *FCGR3* (3.1-fold, *p = 1.4e-06*), and *CSF1R* (1.5-fold, *p = 0.0013*) are significantly increased in the plaque niche compared to non-plaque areas.

Functional studies have begun to identify a role for the TREM2/DAP12 pathway in promoting cell survival and phagocytic capacity [63]. TREM2 deficiency reportedly impairs the phagocytosis of apoptotic neurons in microglia [64], possibly through apolipoprotein E and lipid-sensing mechanisms [65–68]. Similarly, in primary microglia isolated from TREM2 knockout mice, impaired phagocytic capacity of microbeads as well as *Escherichia coli* bacteria imply that TREM2 plays an important regulatory role in innate immune activity and possibly host defense mechanism [69]. Similar functional insights have been reported for CD33. A study by Gricuic et al. observed increased expression of CD33 positive microglia in AD brains, a significant positive correlation between Aβ pathology and CD33 expression as well as an increase in microglial uptake of Aβ in vitro after CD33 inactivation [32]. Interestingly, clinical data show an increase in PiB imaging in patients with the risk allele for CD33 [16], further demonstrating a relationship with amyloid pathology and potential to track the progression of disease-related PET-imaging biomarkers. These findings serve as the foundation for the therapeutic hypothesis that blockade of CD33 function could be a compelling alternative to accelerate Aβ clearance [32]. Thus, when considering collectively the functional roles for TREM2 and CD33 in microglia and AD, it appears that converging and opposing innate immune regulation may exist and dictate risk status for human AD.

## Conclusions

The current study highlights the importance of careful consideration and selection of appropriate APP mouse models specifically for exploring neuroimmune modulation, in particular ITIM/ITAM-associated mechanisms, such as TREM2 and CD33. Combined with the insights from human genetics, these findings provide broad evidence for the presence of a complex relationship between activating and inhibitory mechanisms which collectively may determine innate immune status around the Aβ plaque niche. Future studies utilizing single-cell profiling of microglia in AD mouse models to further characterize the co-expression of immune activating (e.g., ITAM) and suppressing (e.g., ITIM) mechanisms and will help to define how the underlying regulation of mechanisms controlling this microglial innate immune rheostat are shaped by neuropathology. Such studies will also help clarify whether distinct heterogeneous sub-populations of microglia exist around amyloid plaques, or whether competing inhibitory and activating nodes are commonly present within individual plaque-associated microglia. The observations provide tissue-level insights into plaque-associated transcriptional signatures to encourage more fine characterization of cell-specific pathway changes across such broad panels of markers.

## Additional files

Additional file 1: **Figure S1.** Nanostring confirmation of plaque associate selected gene in TgCRND8. Selected plaque associated genes that were upregulated in LCM tissue in TgCRND8 mice by RNAseq were confirmed using a Nanostring customized chip (n=4 per group). Bar graphs show nCounts (mean ± s.e.m) to highlight changes for a subset of specific transcripts included for direct comparison. *p < 0.05, **p < 0.01, by student t-test, 2-tailed. (JPG 51 kb)

Additional file 2: **Figure S2.** Nanostring confirmation of plaque associate selected gene in TgCRND8. Selected plaque associated genes that were upregulated in LCM tissue in TgCRND8 mice by RNA seq confirmed using Nanostring customized chip (n=4 per group). Dot plots show mRNAs transcripts (mean ± s.e.m). *p < 0.05, **p < 0.01, ***p < 0.001, by student t-test, 2-tailed. (JPG 175 kb)

Additional file 3: **Table S1.** Age and strain-related transcriptomic changes in AD models. RNAseq was carried out on cortexes isolated from Tg2576 (ages: 3mo, 6mo, 9mo, 12mo and 15mo, respectively), TgCRND8 (1.5mo, 3mo, 4.5mo, 6mo and 10mo, respectively) and WT age-matched controls mice. Data shows statistical analysis of age correlation between each strain, *p*-value and log10 analysis. Differentially expressed genes were identified by Pearson correlation or T-test using Matlab R2010b (Mathworks). A p-value cutoff of < 0.001 was used to identify differentially expressed genes. The FDR corresponding to this p-value is given in each of the comparisons to convey relative signature confidence. Set annotation analysis was performed by comparing input sets to GeneGo (www.genego.com), Ingenuity (www.ingenuity.com) and KEGG (www.genome.jp/kegg/) pathway sets. Bonferroni corrected hypergeometric *p*-values expectation (*e*)-values) of less than 0.1 were considered significant overlap between sets. (XLS 7280 kb)

Additional file 4: **Table S2.** Age and strain-related pathway analysis in TgCRND8 cortex. Pathways analysis showing data from upregulated network processes in TgCRND8 cortex and plaque-associated tissue. Differentially expressed genes were identified by Pearson correlation or T-test using Matlab R2010b (Mathworks). A *p*-value cutoff of < 0.001 was used to identify differentially expressed genes. The FDR corresponding to this *p*-value is given in each of the comparisons to convey relative signature confidence. Set annotation analysis was performed by comparing input sets to GeneGo (www.genego.com), Ingenuity (www.ingenuity.com) and KEGG (www.genome.jp/kegg/) pathway sets. Bonferroni corrected hypergeometric *p*-values (expectation (*e*)-values) of less than 0.1 were considered significant overlap between sets. (XLSX 81 kb)

Additional file 5: **Figure S3.** Tg2576 brain sections illustrating amyloid-beta pathology. Representative images of plaque pathology in 15-month old transgenic mice via anti-amyloid-β, 1-12 (26D6). (JPG 183 kb)

Additional file 6: **Table S3.** Human AD translational transcriptomic profiling. Comparative gene network analysis was carried out from the mouse models used (tgCRND8 and Tg2576 models) in our study with that of: 1) Human aging (up-regulated) 2) Human aging (down-regulated) 3) Human inflammation signature 3) ITIM/ITAM-domain associated network signature 3) Mouse microglial gene signature (Barres et al., 2013) and 4) Mouse disease-associated microglia or DAM gene signature (Keren-Sheul et al., 2017). Differentially expressed genes were identified by Pearson correlation or T-test using Matlab R2010b (Mathworks). A. *p*-value cutoff of < 0.001 was used to identify differentially expressed genes. The FDR corresponding to this *p*-value is given in each of the comparisons to convey relative signature confidence. Set annotation analysis was performed by comparing input sets to GeneGo (www.genego.com), Ingenuity (www.ingenuity.com) and KEGG (www.genome.jp/kegg/) pathway sets. Bonferroni corrected hypergeometric *p*-values (expectation (*e*)-values) of less than 0.1 were considered significant overlap between sets. (XLSX 26 kb)

Additional file 7: **Table S4.** Laser captured plaque transcriptomic signature. RNAseq data of laser-capture microscopy of TgCRND8 plaques versus non-plaque tissue as outlined in schematic in Fig. 2. Data shows statistical analysis of TgCRND8 plaques versus non-plaque tissue at 6 months of age (*p*-value) and log10 analysis of the ratio of TgCRND8 plaques versus non-plaque tissue. Differentially expressed genes were identified by Pearson correlation or T-test using Matlab R2010b (Mathworks). A *p*-value cutoff of < 0.001 was used to identify differentially expressed genes. The FDR corresponding to this *p*-value is given in each of the comparisons to convey relative signature confidence. Set annotation analysis was performed by comparing input sets to GeneGo (www.genego.com), Ingenuity (www.ingenuity.com) and KEGG (www.genome.jp/kegg/) pathway sets. Bonferroni corrected hypergeometric *p*-values (expectation (*e*)-values) of less than 0.1 were considered significant overlap between sets. (XLS 1410 kb)

Additional file 8: **Figure S4.** Expression of microglial versus peripheral macrophage markers. Data were analyzed for fold changes in genes associated with either resident microglia (A) or peripheral macrophages (B) as outlined by Hickman et al., 2013. Results demonstrate significant (*p < 0.001) expression of 7 out of the top 25 most abundant genes in microglia in plaque samples compared to non-plaque controls whereas only 1 gene, *C4b*, of genes associated with peripheral macrophages displayed significant expression in plaque compared to non-plaque samples. For whole cortex samples, half of the top 25 microglial genes were significantly increased in plaque samples compared to non-plaque. (JPG 43 kb)

## Abbreviations

AD: Alzheimer’s disease
APP: Amyloid precursor protein
Aβ: Amyloid-beta
CNS: Central nervous system
DAM: Disease-associated microglia
GWAS: Genome-wide association studies
ITAM: Immunoreceptor tyrosine-based activation motif
ITIM: Immunoreceptor tyrosine-based inhibition motif
LOAD: Late-onset Alzheimer’s disease
