# Supplementary figures and images for: Human Alzheimer’s disease gene expression signatures and immune profile in APP mouse models: a discrete transcriptomic view of Aβ plaque pathology
Source: J Neuroinflammation. 2018 Sep 6;15:256. doi: 10.1186/s12974-018-1265-7 (PMC6127905; doi:10.1186/s12974-018-1265-7)

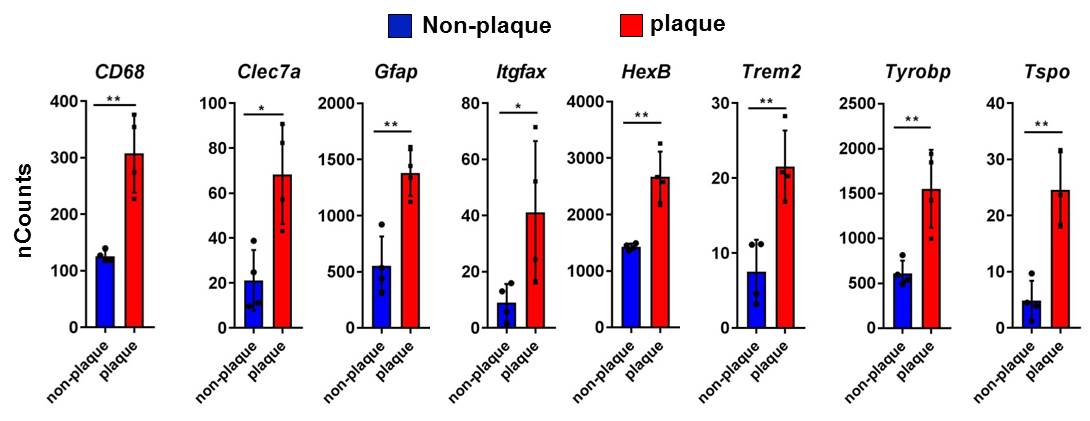

Supplement: Supplementary file 1 — Figure S1. Nanostring confirmation of plaque associate selected gene in TgCRND8. Selected plaque associated genes that were upregulated in LCM tissue in TgCRND8 mice by RNAseq were confirmed using a Nanostring customized chip (n=4 per group). Bar graphs show nCounts (mean ± s.e.m) to highlight changes for a subset of specific transcripts included for direct comparison. *p < 0.05, **p < 0.01, by student t-test, 2-tailed. (JPG 51 kb) [file 12974_2018_1265_MOESM1_ESM.jpg]

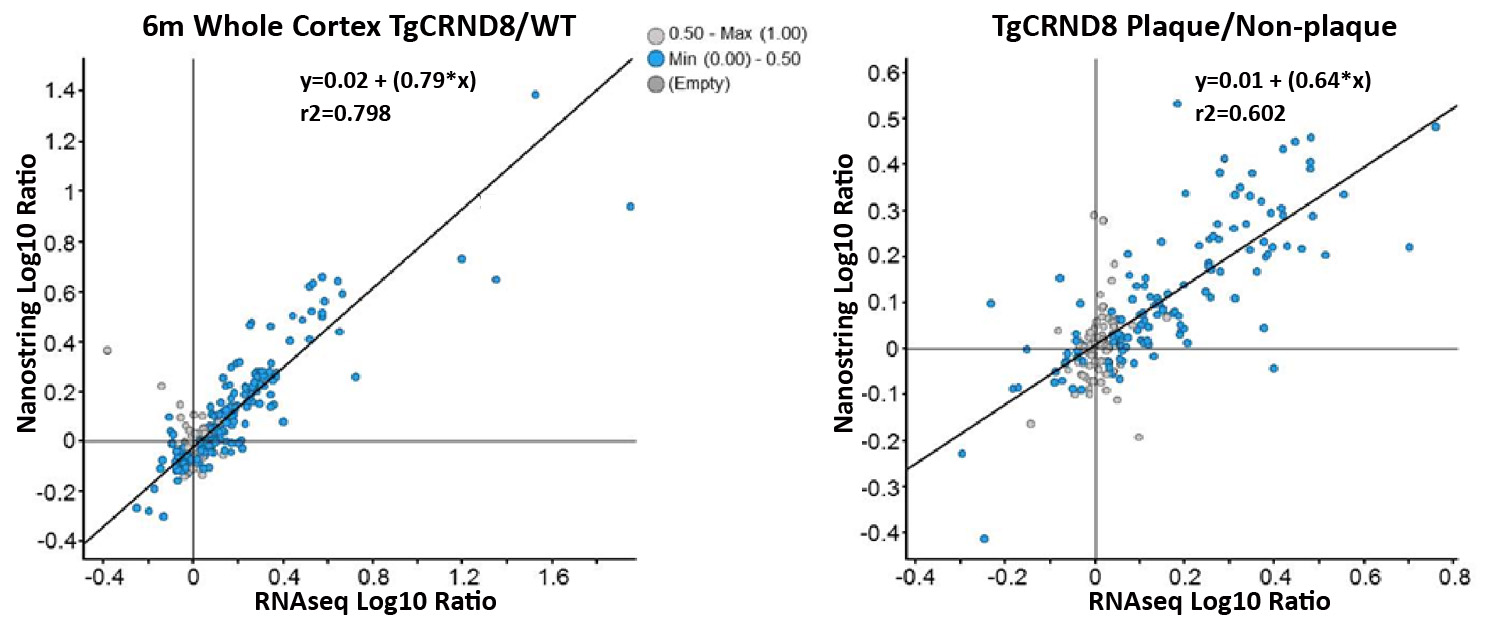

Supplement: Supplementary file 2 — Figure S2. Nanostring confirmation of plaque associate selected gene in TgCRND8. Selected plaque associated genes that were upregulated in LCM tissue in TgCRND8 mice by RNA seq confirmed using Nanostring customized chip (n=4 per group). Dot plots show mRNAs transcripts (mean ± s.e.m). *p < 0.05, **p < 0.01, ***p < 0.001, by student t-test, 2-tailed. (JPG 175 kb) [file 12974_2018_1265_MOESM2_ESM.jpg]

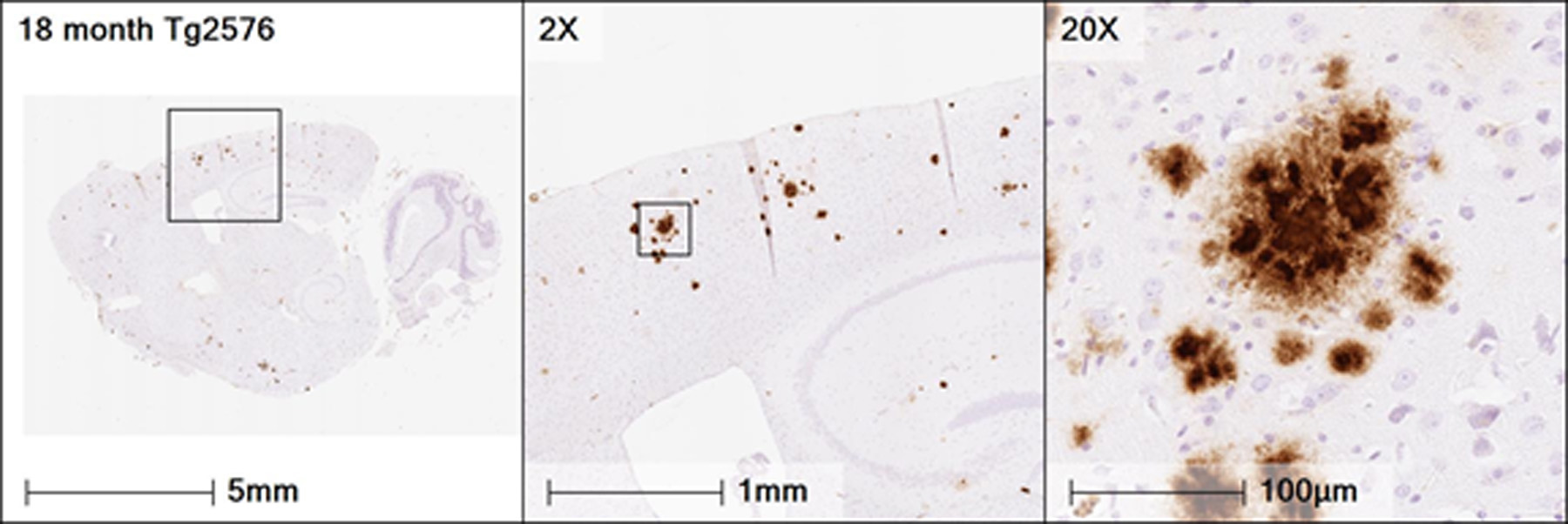

Supplement: Supplementary file 5 — Figure S3. Tg2576 brain sections illustrating amyloid-beta pathology. Representative images of plaque pathology in 15-month old transgenic mice via anti-amyloid-β, 1-12 (26D6). (JPG 183 kb) [file 12974_2018_1265_MOESM5_ESM.jpg]

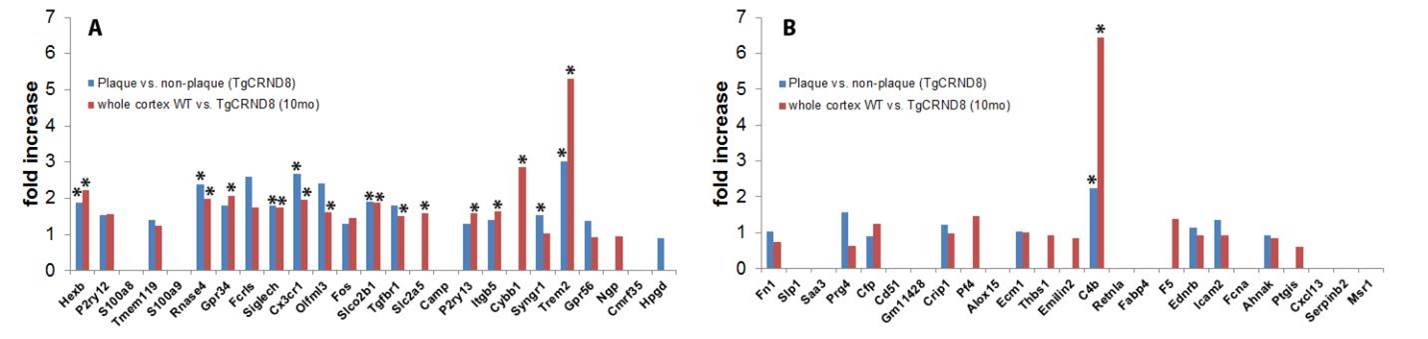

Supplement: Supplementary file 8 — Figure S4. Expression of microglial versus peripheral macrophage markers. Data were analyzed for fold changes in genes associated with either resident microglia (A) or peripheral macrophages (B) as outlined by Hickman et al., 2013. Results demonstrate significant (*p < 0.001) expression of 7 out of the top 25 most abundant genes in microglia in plaque samples compared to non-plaque controls whereas only 1 gene, C4b, of genes associated with peripheral macrophages displayed significant expression in plaque compared to non-plaque samples. For whole cortex samples, half of the top 25 microglial genes were significantly increased in plaque samples compared to non-plaque. (JPG 43 kb) [file 12974_2018_1265_MOESM8_ESM.jpg]
